# Supplementary material for: A thermophilic chemolithoautotrophic bacterial consortium suggests a mutual relationship between bacteria in extreme oligotrophic environments
Source: Commun Biol. 2023 Mar 1;6:230. doi: 10.1038/s42003-023-04617-4 (PMC9977764; doi:10.1038/s42003-023-04617-4)
Supplement: Supplementary file 2 — Supplementary Information [file 42003_2023_4617_MOESM2_ESM.pdf]

Supplementary Information

A thermophilic chemolithoautotrophic bacterial consortium suggests a mutual relationship between bacteria in extreme oligotrophic environments

Yuri Pinheiro [1+], Fabio Faria da Mota [2+], Raquel Peixoto [3,4], Jan Dirk van Elsas [5], Ulysses Lins [1], Jorge L. Mazza Rodrigues [6], and Alexandre Soares Rosado [3,4,7,*]

[1]Institute of Microbiology, Federal University of Rio de Janeiro, Rio de Janeiro, Brazil.
[2]Computational and Systems Biology Laboratory, Oswaldo Cruz Institute, FIOCRUZ, Rio de Janeiro, Brazil.
[3]Red Sea Research Center (RSRC), King Abdullah University of Science and Technology (KAUST), Thuwal, 23955-6900, Saudi Arabia.
[4]Computational Bioscience Research Center (CBRC), King Abdullah University of Science and Technology (KAUST), Thuwal, 23955-6900, Saudi Arabia.
[5]Microbial Ecology, Groningen University, Groningen, The Netherlands.
[6] Department of Land, Air, and Water Resources, University of California Davis, Davis, USA.
[7]Bioscience Program, Biological and Environmental Sciences and Engineering Division (BESE), King Abdullah University of Science and Technology (KAUST), Thuwal, Saudi Arabia.

[*]Corresponding author: alexandre.rosado@kaust.edu.sa

a

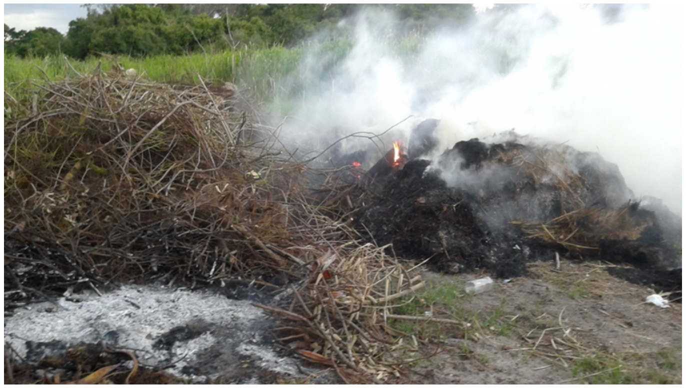

b

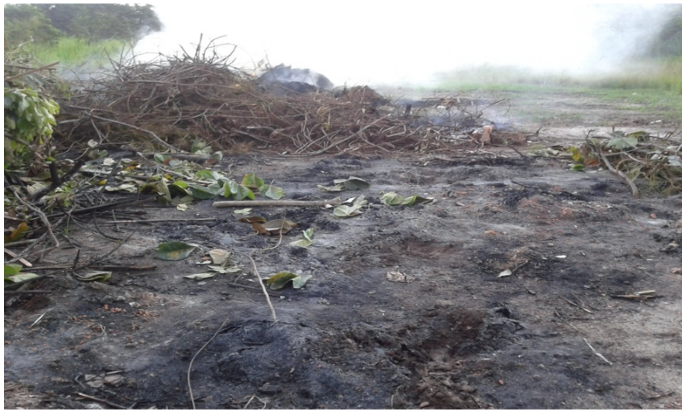

Parameter	Methods	Unit	Control Soil	Burned soil
pH	(H_2O)	NA	7.4	8.1
Total N	Dumas	%	0.075	0.059
Organic C	(Oxi-Red)	%	1.5	1.8
M.O	(Oxi-Red)	dag/kg^2	2.5	3.1
C/N Ratio	NA	NA	19.5	30.7
P	(Mehlich-1)	mg/dm^3	346	977
K	(Mehlich-1)	mg/dm^3	44	778
Na	(Mehlich-1)	$Cmolc/dm^3$	0.21	0.29
Ca	(KCl 1 mol/L)	$Cmolc/dm^3$	6.9	5.7
Mg	(KCl 1 mol/L)	$Cmolc/dm^3$	1.8	2.7
K	(KCl 1 mol/L)	$Cmolc/dm^3$	0.11	1.99
Al	(KCl 1 mol/L)	$Cmolc/dm^3$	0	0
H+Al	Ca Acetate	$Cmolc/dm^3$	0.4	0.1
S	Sum of bases	$Cmolc/dm^3$	9	10.7
T	(C.T.C.)	$Cmolc/dm^3$	9	10.8
V%	Saturation of bases	$Cmolc/dm^3$	95.6	99
m	Al saturation	%	0	0
n	Na saturation	%	2.2	2.7
t	effectiv C.T.C.	%	9	10.7

Supplementary Figure 1. Field site where the samples were collected.

Soil samples were collected from under the pile of burned vegetal material. Images of the typical burning process in the sampling site (a and b) and a table showing the soil characterization under the vegetal ashes and in a control soil with no history of burning collected nearby.

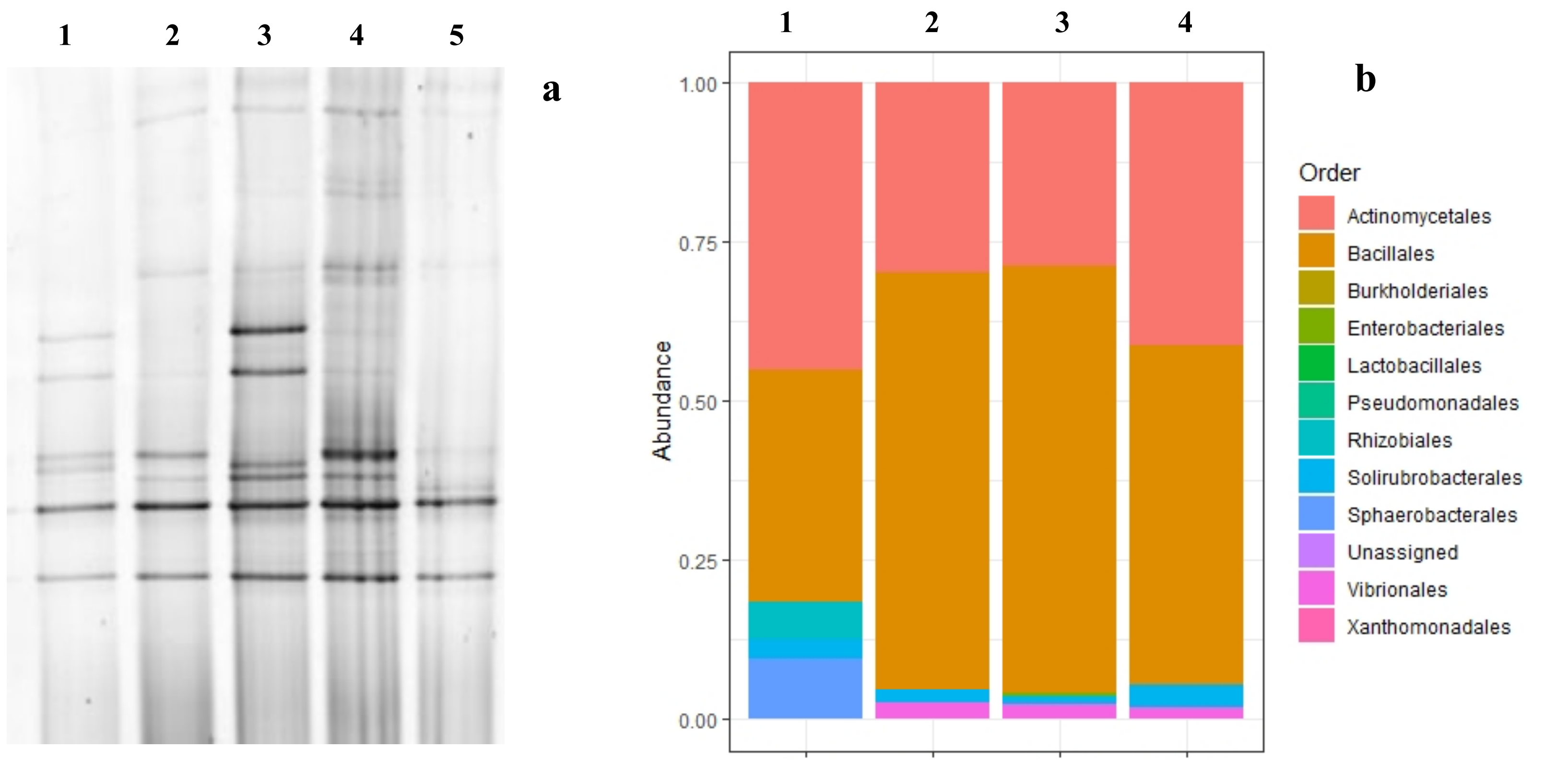

Supplementary Figure 2. Stability of the Carbonitroflex (CNF) consortium.

a) Denaturing gradient gel electrophoresis (6% acrylamide gel containing a urea and formamide gradient [range: 35%–65%] run at 70 V for 16 h) of *rrs* gene fragments from samples collected from the consortium over time. Transfers occur each 15 days. (1) After 5 transfers; (2) after 12 transfers; (3) after 16 transfers; (4) after 36 transfers; and (5) after 44 transfers (approximately 3 years). b) Bar plot showing the taxonomic composition of the carbonitroflex consortium obtained via *rrs* gene amplicon sequencing (Illumina) in the year of its isolation (1) and after consecutive transfers (i.e., after 1 year (2), 2 years (3) and 3 years (4).
