# Supplementary material for: A thermophilic chemolithoautotrophic bacterial consortium suggests a mutual relationship between bacteria in extreme oligotrophic environments
Source: Commun Biol. 2023 Mar 1;6:230. doi: 10.1038/s42003-023-04617-4 (PMC9977764; doi:10.1038/s42003-023-04617-4)
Supplement: Supplementary file 3 — Description of Additional Supplementary Files [file 42003_2023_4617_MOESM3_ESM.pdf]

Description of Additional Supplementary Files

File name: Supplementary Data 1
Description: The source data behind the graphs in the paper. Measurements were made with the Digital Micrograph software (Gatan, Inc.).

File name: Supplementary Data 2
Description: Percentage of annotated protein coding genes.
