# Supplementary material for: A thermophilic chemolithoautotrophic bacterial consortium suggests a mutual relationship between bacteria in extreme oligotrophic environments
Source: Commun Biol. 2023 Mar 1;6:230. doi: 10.1038/s42003-023-04617-4 (PMC9977764; doi:10.1038/s42003-023-04617-4)
Supplement: Supplementary file 6 — Reporting Summary [file 42003_2023_4617_MOESM6_ESM.pdf]

nature portfolio

Corresponding author(s): Alexandre Soares Rosado

Last updated by author(s): 09/02/2023

Reporting Summary

Nature Portfolio wishes to improve the reproducibility of the work that we publish. This form provides structure for consistency and transparency in reporting. For further information on Nature Portfolio policies, see our Editorial Policies and the Editorial Policy Checklist.

Statistics

For all statistical analyses, confirm that the following items are present in the figure legend, table legend, main text, or Methods section.

n/a	Confirmed	
☐	☒	The exact sample size (*n*) for each experimental group/condition, given as a discrete number and unit of measurement
☐	☒	A statement on whether measurements were taken from distinct samples or whether the same sample was measured repeatedly
☐	☒	The statistical test(s) used AND whether they are one- or two-sided<br>*Only common tests should be described solely by name; describe more complex techniques in the Methods section.*
☐	☒	A description of all covariates tested
☐	☒	A description of any assumptions or corrections, such as tests of normality and adjustment for multiple comparisons
☒	☐	A full description of the statistical parameters including central tendency (e.g. means) or other basic estimates (e.g. regression coefficient) AND variation (e.g. standard deviation) or associated estimates of uncertainty (e.g. confidence intervals)
☒	☐	For null hypothesis testing, the test statistic (e.g. *F*, *t*, *r*) with confidence intervals, effect sizes, degrees of freedom and *P* value noted<br>*Give P values as exact values whenever suitable.*
☒	☐	For Bayesian analysis, information on the choice of priors and Markov chain Monte Carlo settings
☐	☒	For hierarchical and complex designs, identification of the appropriate level for tests and full reporting of outcomes
☒	☐	Estimates of effect sizes (e.g. Cohen's *d*, Pearson's *r*), indicating how they were calculated

Our web collection on statistics for biologists contains articles on many of the points above.

Software and code

Policy information about availability of computer code

Data collection	No software was used

Data analysis	All images were processed with Adobe Photoshop CS5 (Adobe System Co., USA). Softwares for genomics and metagenomics analysis: Barrnap (https://github.com/tseemann/barrnap) ribosomal predictor software, MEGAN (Huson et al. 2011). The sequences of rpoB, dnak, and atpD were assembled into contigs using the SPAdes software (Bankevich et al. 2012) and compared using the Basic Local Alignment Search tool (BLAST) from NCBI (rpoB, dnak, and atpD) and Seq Match from the Ribosomal Database Project database (rrs—only sequences derived from culturable microorganisms and with a size >1200 bp). The five most similar matches were selected for each contig and each gene and aligned using the Multiple Sequence Comparison by Log-Expectation software (version 3.8.31). Phylogeny reconstruction for each gene was performed with MEGA-7 (Kumar, Stecher, and Tamura 2016). SPAdes software 3.8.0 (Bankevich et al., 2012). The BLAST Ring Image Generator (BRIG) software (Alikhan et al., 2011, version 0.95-dev.0004) was used to display circular comparisons between the MAGs from the consortium and the NCBI reference genomes. Alignments were generated by BRIG using the BLASTN program and the concatenated contigs of MAGs (this study) or the previously isolated LEMMY01 (Souza et al., 2017) and the reference strain sequences (UBT1, H1, DMS18167, DSM20745, and DSM13240). Additionally, the DNAPlotter software (Carver et al., 2009, version 17) was used to highlight the GC content plot.Overlapping paired-end reads were merged using the software Paired-End reAd merger (Zhang et al., 2014). Merged and unmerged reads were mapped to MAGs using the software Bowtie2 (Langmead & Salzberg, 2012) to extract the reads belonging to each member, including the missing genes in the reference genomes. The extracted reads were aligned to the NCBI nr database using BlastX. Metabolic reconstructions for the consortium members were obtained using the KEGG database and MEGAN5 (Huson et al., 2011). Differences between metabolic pathways, heteromultimeric enzymes, and transporters were highlighted to determine the possibility of metabolic complementation among consortium members. The completeness of MAGs was evaluated using the CheckM software (Parks et al., 2015), and the complementation between the main metabolic pathways was assessed by submitting the MAGs and the published whole-genome sequencing (WGS) data of Geobacillus LEMMY01 (Souza et al., 2017) into the tool metabolisHMM (McDaniel et al., 2019).

For manuscripts utilizing custom algorithms or software that are central to the research but not yet described in published literature, software must be made available to editors and reviewers. We strongly encourage code deposition in a community repository (e.g. GitHub). See the Nature Portfolio guidelines for submitting code & software for further information.

Data

Policy information about availability of data

All manuscripts must include a data availability statement. This statement should provide the following information, where applicable:

- Accession codes, unique identifiers, or web links for publicly available datasets
- A description of any restrictions on data availability
- For clinical datasets or third party data, please ensure that the statement adheres to our policy

All data generated or analysed during this study are included in this published article (and its supplementary information files).

Human research participants

Policy information about studies involving human research participants and Sex and Gender in Research.

Reporting on sex and gender	N/A
Population characteristics	N/A
Recruitment	N/A
Ethics oversight	N/A

Note that full information on the approval of the study protocol must also be provided in the manuscript.

Field-specific reporting

Please select the one below that is the best fit for your research. If you are not sure, read the appropriate sections before making your selection.

☒ Life sciences ☐ Behavioural & social sciences ☐ Ecological, evolutionary & environmental sciences

For a reference copy of the document with all sections, see nature.com/documents/nr-reporting-summary-flat.pdf

Life sciences study design

All studies must disclose on these points even when the disclosure is negative.

Sample size	No sample size was necessary, because this sytudy was based on obtaining an unique bacterial consortium.
Data exclusions	No data were excluded from the analysis.
Replication	All experiments were done in replicates (3 or more) and information was detailed in the text. All attempts of replication were sucessful.
Randomization	Randomization was not necessary. The study was based on a natural enriched autotrophic consortium.

Blinding	Blinding was not relevant for this study. This research is based on detailed observations and a model of interaction among the bacterial consortiummembers.

Reporting for specific materials, systems and methods

We require information from authors about some types of materials, experimental systems and methods used in many studies. Here, indicate whether each material, system or method listed is relevant to your study. If you are not sure if a list item applies to your research, read the appropriate section before selecting a response.

Materials & experimental systems

n/a	Involved in the study
☒	☐ Antibodies
☒	☐ Eukaryotic cell lines
☒	☐ Palaeontology and archaeology
☒	☐ Animals and other organisms
☒	☐ Clinical data
☒	☐ Dual use research of concern

Methods

n/a	Involved in the study
☒	☐ ChIP-seq
☒	☐ Flow cytometry
☒	☐ MRI-based neuroimaging
